# Grading of IDH-mutant astrocytoma using diffusion, susceptibility and perfusion-weighted imaging

**DOI:** 10.1186/s12880-022-00832-3

**Published:** 2022-05-29

**Authors:** Xiefeng Yang, Zhen Xing, Dejun She, Yu Lin, Hua Zhang, Yan Su, Dairong Cao

**Affiliations:** 1 Department of Radiology, First Affiliated Hospital of Fujian Medical University, 20 Cha-Zhong Road, Fuzhou, 350005 Fujian People’s Republic of China; 2 Fujian Key Laboratory of Precision Medicine for Cancer, The First Affiliated Hospital, Fujian Medical University, Fuzhou, 350005 People’s Republic of China; 3 Key Laboratory of Radiation Biology of Fujian Higher Education Institutions, The First Affiliated Hospital, Fujian Medical University, Fuzhou, 350005 People’s Republic of China

**Keywords:** IDH-mutant, Astrocytoma, SWI, DSC-PWI

## Abstract

**Background:**

The accurate grading of IDH-mutant astrocytoma is essential to make therapeutic strategies and assess the prognosis of patients. The purpose of this study was to investigate the usefulness of DWI, SWI and DSC-PWI in grading IDH-mutant astrocytoma.

**Methods:**

One hundred and seven patients with IDH-mutant astrocytoma who underwent DWI, SWI and DSC-PWI were retrospectively reviewed. Minimum apparent diffusion coefficient (ADC_min_), intratumoral susceptibility signal intensity(ITSS) and maximum relative cerebral blood volume (rCBV_max_) values were assessed. ADC_min_, ITSS and rCBV_max_ values were compared between grade 2 vs. grade 3, grade 3 vs. grade 4 and grade 2 + 3 vs. grade 4 tumors. Logistic regression, tenfold cross-validation,and receiver operating characteristic (ROC) curve analyses were used to assess their diagnostic performances.

**Results:**

Grade 4 IDH-mutant astrocytomas showed significantly lower ADC_min_ and higher rCBV_max_ as compared to grade 3 tumors (adjusted *P* < 0.001). IDH-mutant grade 3 astrocytomas showed significantly lower ITSS levels as compared with grade 4 tumors (adjusted *P* < 0.001). ITSS levels between IDH-mutant grade 2 and grade 3 astrocytomas were significantly different (adjusted *P* = 0.002). Combined the ADC_min_, ITSS and rCBV_max_ resulted in the highest AUC for differentiation grade 2 and grade 3 tumors from grade 4 tumors.

**Conclusion:**

ADC_min,_ rCBV_max_ and ITSS can be used for grading the IDH-mutant astrocytomas. The combination of ADC_min,_ ITSS and rCBV_max_ could improve the diagnostic performance in grading of IDH-mutant astrocytoma.

## Background

According to the 2021 World Health Organization (WHO) central nervous system (CNS) classification system, IDH-mutant astrocytomas were divided into three categories: grade 2, IDH-mutant astrocytoma, grade 3, IDH-mutant astrocytoma and grade 4, IDH-mutant astrocytoma [1]. Previous studies revealed that prognosis of IDH-mutant astrocytomas significantly varied according to their grade [2, 3]. The prognosis of grade 4, IDH-mutant astrocytoma, compared with its lower grade counterparts, showed a dismal outcome. Thus, a preoperative grading of these entities could be helpful in patients’ therapeutics and prognostics, especially in patients with contraindication to operation or in cases with unresectable lesions [4].

Diffusion-weighted imaging (DWI) could noninvasively assess the Brownian movement of water molecules in vivo, and indirectly reflect the cellularity of cerebral tumors by means of apparent diffusion coefficient (ADC) values [5]. Dynamic susceptibility contrast perfusion-weighted imaging (DSC-PWI) could provide hemodynamic parameters with tumor angiogenesis and may be useful for glioma grading [6]. As another advanced technique, susceptibility-weighted imaging (SWI) can semi-quantitatively assess tumor micro-hemorrhages and vasculature through intratumoral susceptibility signal intensity (ITSS) [7]. All these methods have been reported differentiation between various grades of gliomas [8–11].

However, few studies focus on grading of IDH-mutant astrocytic gliomas. Therefore, we determined to focus on our study about IDH-mutant astrocytic gliomas only, according to the updated WHO classification. We hypothesized that advanced imaging techniques including DWI, SWI, and DSC-PWI might be useful for grading of IDH-mutant astrocytic gliomas, and the combination of DWI, DSC-PWI, and SWI may contribute to improve the accuracy of diagnosis in IDH-mutant astrocytic gliomas.

## Methods

### Patients

This retrospective study was approved by the ethics committee of our hospital and patients' informed consent was waived. Patients from July 2014 to March 2020 were reviewed at our hospital. The inclusion criteria were the following: (1) IDH-mutant grade 2–4 astrocytomas confirmed by histopathology according to the 2021 WHO classification criteria; (2) a pretreatment MRI scans included conventional MRI(cMRI), DWI, SWI and DSC-PWI. The exclusion criteria were as follows: (1) motion artifacts; (2) poor images quality. A total of 107 patients, including 56 patients with grade 2 IDH mutant astrocytoma, 29 patients with grade 3 IDH mutant astrocytoma, and 22 patients with grade 4 IDH mutant astrocytoma, were enrolled in this study. Of these patients, 63 were male and 44 were female. The age of patients ranged from 18 to 68 years, with a mean age of 40.39 years.

### MRI Protocol

Imaging was performed with 3.0 T MR systems (Magnetom Skyra or Verio; Siemens, Erlangen, Germany). The cMRI sequences included T2WI, FLAIR, T1WI, and contrast-enhanced T1WI. All cMRI sequences were performed with a field of view (FOV) = 220 mm × 220 mm, slices = 20, slice thickness = 5 mm and interslice gap = 1 mm. The total scan time of T2WI, FLAIR, T1WI, and contrast-enhanced T1WI were 72 s, 66 s,136 s, and 66 s, respectively.

DWI was performed with a single-shot echo planar imaging sequence. The b-values were 0 and 1000 s/mm2 with diffusion gradients encoded in the 3 orthogonal directions to generate 3 sets of diffusion-weighted images. The ADC map was generated automatically by the MR imaging system. The imaging parameters were as follows: FOV = 220 mm × 220 mm, repetition time/echo time (TR/TE) = 8200/102 ms; NEX = 2.0; slices = 20; slice thickness = 5 mm; interslice gap = 1 mm. The total scan time was 1 min 32 s.

SWI was performed by 3D fully flow-compensated gradient echo sequence, and a reconstruction by combining the magnitude and phase images. SWI map was generated automatically on the MR imaging scanner. The parameters were as follows: FOV = 220 mm × 220 mm;TR/TE = 27/20 ms; flip angle(FA) = 15°; NEX = 1; slices = 60; slice thickness = 2.0 mm; intersection gap = 0.4 mm. The total scan time was 2 min 19 s.

DSC-PWI was obtained with a gradient-recalled T2*-weighted echo-planar imaging sequence. The imaging parameters were as follows: FOV = 220 mm × 220 mm; TR/TE = 1000–1250/54 ms; FA = 35°; NEX = 1.0; slices = 20; slice thickness/intersection gap = 5 mm/1 mm. When the scan was to the fourth phase of DSC-PWI, 0.1 mmol/kg body weight of gadopentetate dimeglumine was injected with an MR-compatible power injector at a flow rate of 5 ml/s through an intravenous catheter, followed by a 20 ml continuous saline flush. The series of 20 sections, 60 phases, and 1200 images were acquired in 1 min 36 s. T2* weighted signal was converted to ∆R2* for CBV processing. The whole-brain CBV maps were obtained by using a single-compartment model, T1 extravasation correction, and an arterial input function. The anterior or medial cerebral artery was identified manually for the arterial input function.

### Image analysis

For the analysis of DWI data, five non-overlapping small round regions of interest (ROIs) were carefully placed in the tumor of the ADC maps to obtained minimum ADC values (ADC_min_) with visual inspection [12]. The ROIs (20-40mm^2^) were placed in the solid part of the lesion (defined on T2WI, SWI and contrast-enhanced T1WI), avoiding necrotic, cystic, hemorrhagic, or apparent blood vessel regions that might affect the measurements of ADC values.

Minimum intensity projection (MIP) technique was conducted for the semi-quantitative assessment of SWI. ITSS scores were evaluated in consensus by two neuroradiologists (with 13 and 5 years of experience, respectively) who blinded to tumor histopathology. The degree of ITSSs contained four grades as previously described [7]: (1) grade 0 = no ITSS; (2) grade 1 = 1–5 dot-like or linear ITSSs; (3) grade 2 = 6–10 dot-like or linear ITSSs; and (4) grade 3 ≥ 11 dot-like or linear ITSSs.

For the evaluation of DSC-PWI data, five non-overlapping round ROIs were placed in the tumor on the CBV map to obtained maximum CBV (CBV_max_). When the rCBV ROIs were drawn, an attempt was made to place them in regions where the rCBV appeared highest. To minimize variances, the relative CBV_max_ (rCBV_max_) was measured by dividing the tumor CBV_max_ by the mean CBV of the contralateral normal appearing white matter. Measurements of rCBVmax values were performed with the same ROIs as those used for ADC measurements. The ROIs for the ADC and rCBV measurements were not identical and were not from the same region of the tumor in each patient. ADC_min_ and rCBV_max_ values were obtained by another neuroradiologist (with 7 years of experience) who blinded to tumors histopathologic data..

### Statistical analysis

Initially, Kolmogorov–smirnov test was used for normality of ADCmin and rCBV values. Kruskall-Wallis one way analysis of variance (ANOVA) was used to seek the difference of ADC_min_ and rCBV_max_ values between the three grades of the tumor. Comparisons of ADC_min_ and rCBV values among different grade IDH-mutant astrocytomas were made with student’s *t*-test. Interobserver variabilities of ITSSs levels were analyzed by kappa statistics. Fisher’s exact test was used to assess ITSS scores among different grade IDH-mutant astrocytomas. Bonferroni correction was performed for multiple comparisons of ADC_min_, ITSS, and rCBV, and the corrected p values should be less than 0.0125.A simple linear regression analysis was performed to calculate the Spearman’s correlation coefficient between ADC_min_, ITSS, rCBV_max_ and tumor grades.

Logistic regression analysis was used to assess the diagnostic effect of total parameters from the DWI,SWI and DSC-PWI model. The receiver operating characteristic (ROC) analysis curves with tenfold cross-validation were obtained to determine the optimal cutoff value for differentiating grade 2,3 and 4 IDH-mutant astrocytoma. For each logistic regression model, cross-validation was performed with a tenfold procedure. The optimal cutoff value was determined as the point in the upper left-hand corner that maximized the sum of the sensitivity and specificity. In addition, comparisons of area under the ROC curve (AUC) for different quantitative variables were made with Z test proposed by DeLong et al. [13].

Statistical analysis was conducted by Statistical Package for the Social Sciences(version 19.0.0, Inc.,Chicago, IL, USA), R, Version R 4.1.2 (R Project for Statistical Computing,http://www.r-project.org), and MedCalc (version 12.1.0. Inc., Mariakerke, Belgium). *P* value less than 0.05 was statistically significant.

## Results

**Fig. 1:**
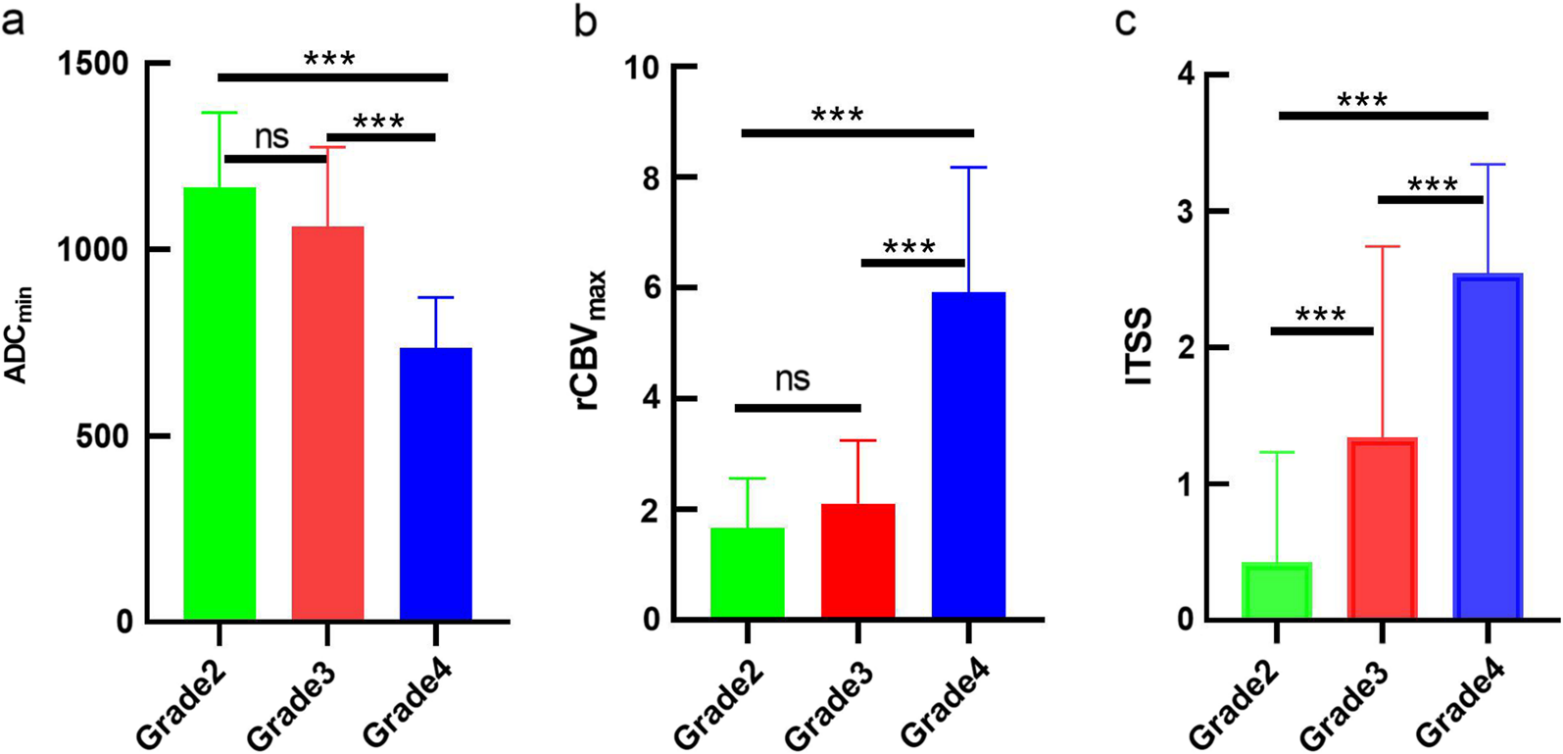
Bar graph demonstrated mean values of (**a**) ADC_min_, (**b**) rCBV_max_ values and (**c**) ITSS among three grades of IDH-mutant astrocytoma. *Indicates *p* value < 0.05, **Indicates *p* value < 0.01, ***Indicates *p* value < 0.001

**Fig. 2:**
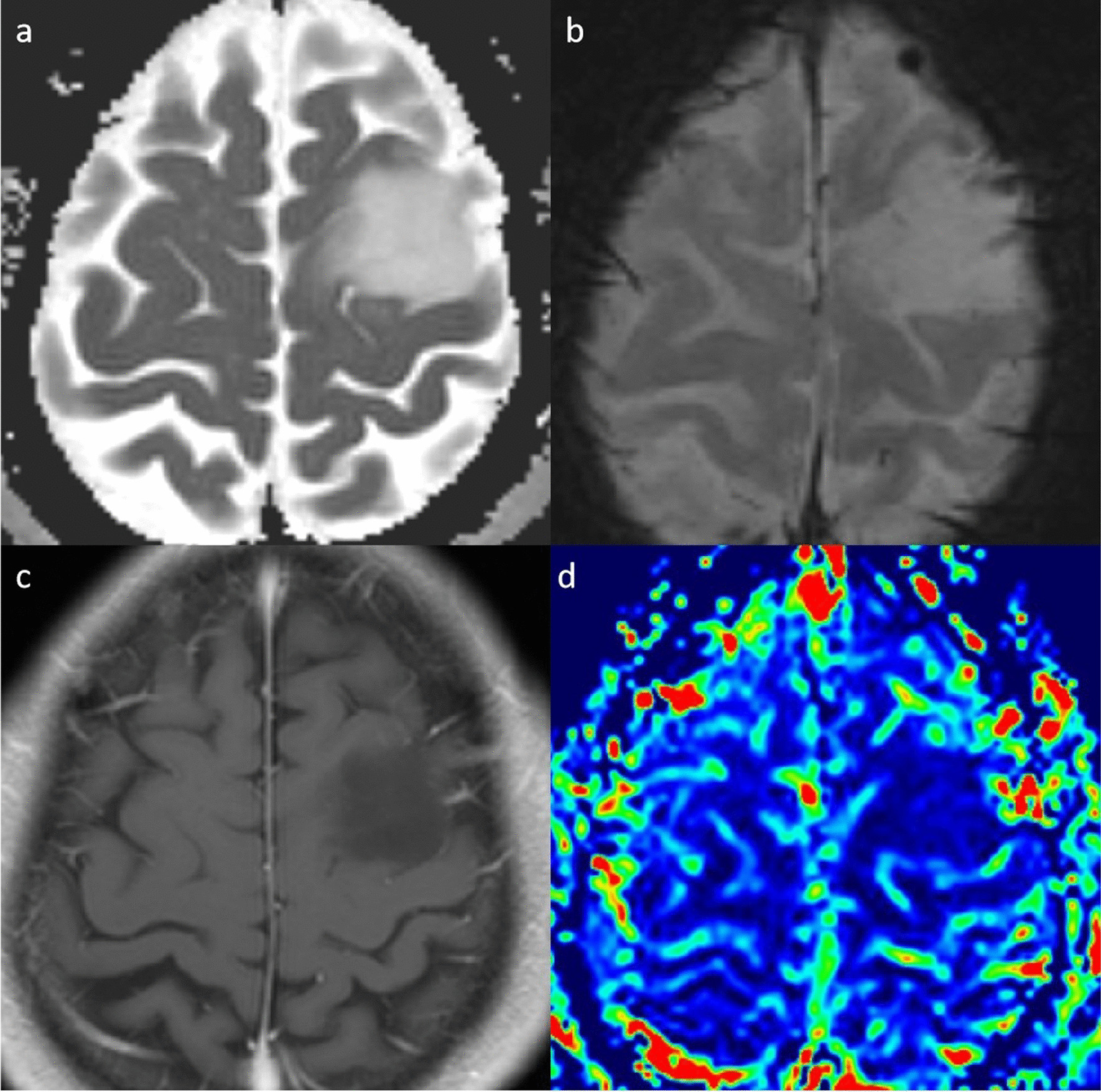
A 40-year-old man with grade 2 IDH-mutant astrocytoma. **a**. ADC map shows an increased ADC value in the lesion (ADC_min_ = 1.645 × 10^−3^mm^2^/s). **b**. SWI map shows no susceptibility signals. **c**. Post-contrast T1WI shows a nonenhancing lesion.** d**. rCBV map shows relatively low the rCBV_max_ value (0.76)

**Fig. 3:**
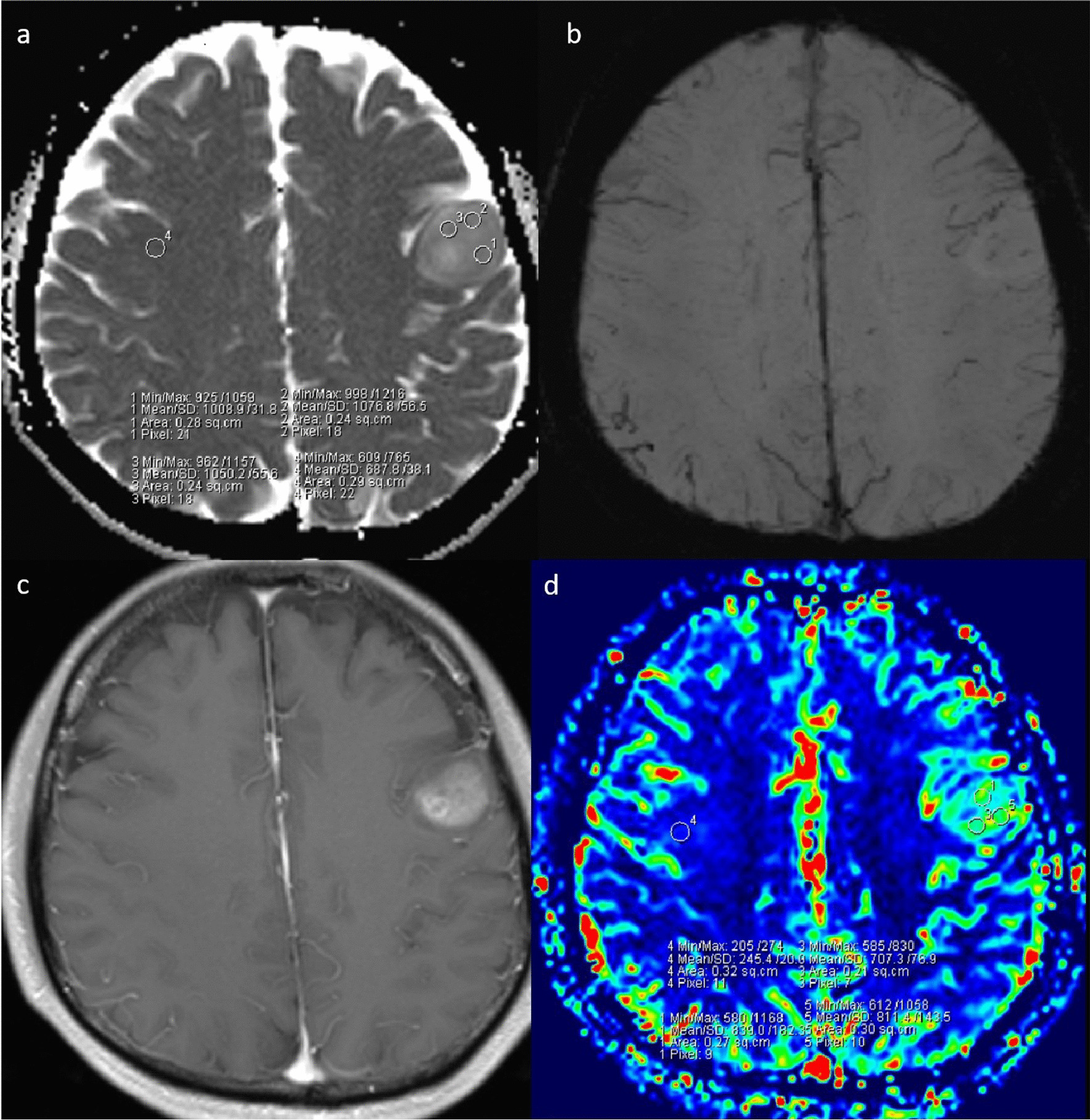
A 53-year-old woman with grade 3 IDH-mutant astrocytoma. **a**. ADC map shows an increased ADC value in the lesion (ADC_min_ = 1.016 × 10^−3^mm^2^/s). **b**. SWI shows obvious ITSS in the left parietal region. **c**. Post-contrast T1WI demonstrates a nonenhancing lesion. **d**. rCBV map shows the rCBV_max_ value of 1.19

**Fig. 4:**
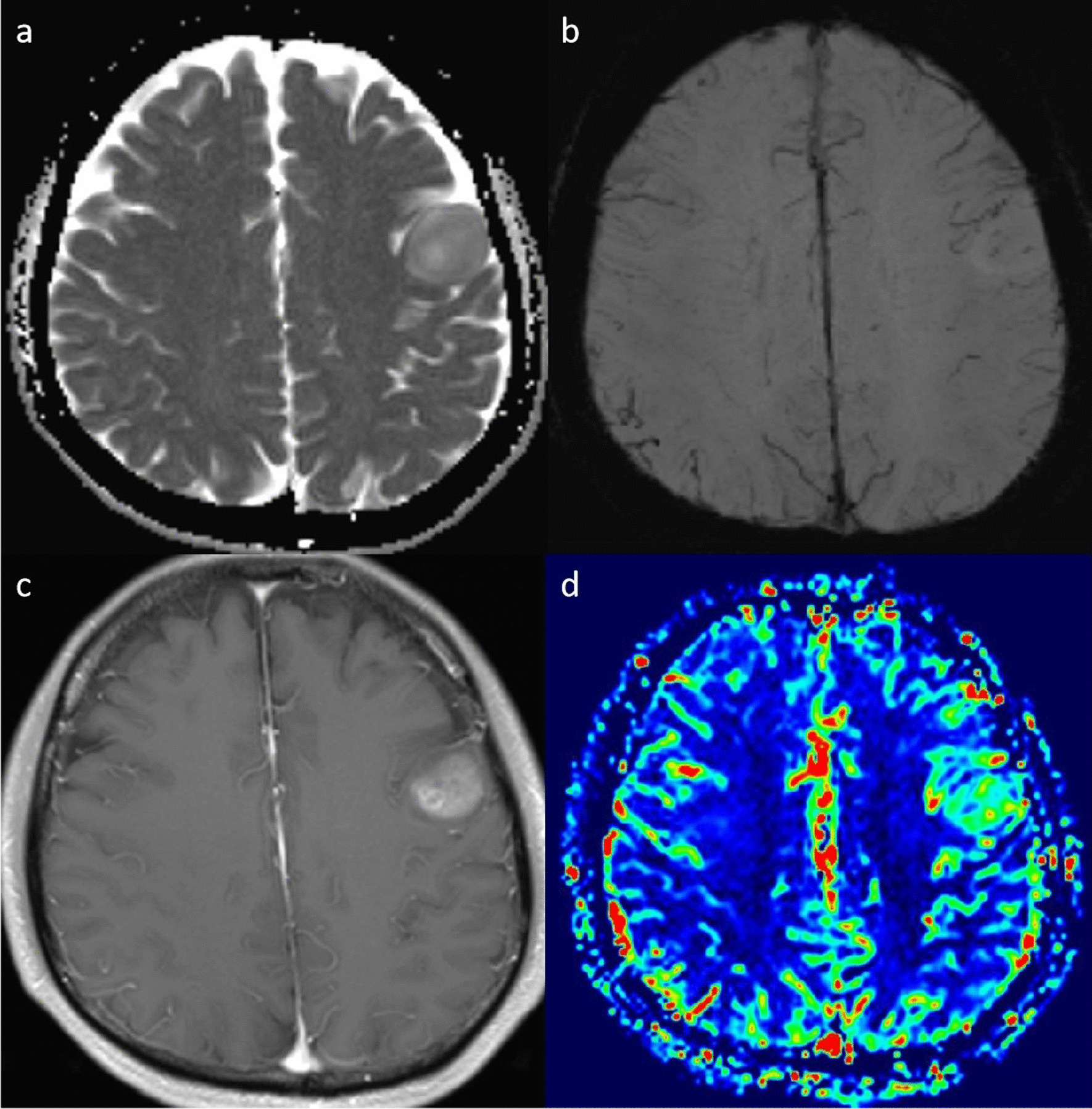
A 43-year-old man with grade 4 IDH-mutant astrocytoma. **a**. ADC map shows a decreased ADC value in the lesion (ADC_min_ = 0.916 × 10^−3^mm^2^/s). **b**. SWI shows medium susceptibility signal. **c**. Post-contrast T1WI shows markedly nodular contrast enhancement. **d**. rCBV map demonstrates relatively high perfusion with the rCBV value of 4.68

The interobserver agreement of ITSS was excellent (Kappa coefficients = 0.858). The ADC_min_, ITSS and rCBV_max_ values of tumors with various grades were summarized in Table 1–2 and Fig. 1. Figures 2, 3 and 4 showed the cases of IDH-mutant astrocytomas with different grades. ADC_min_ and rCBV_max_ values were significant different between grade 2 + 3 and grade 4 IDH-mutant astrocytomas, and between grade 3 and grade 4 IDH-mutant astrocytomas (both adjusted *P* < 0.001). while ITSS scores were significant differentbetween grade 2 and grade 3 IDH-mutant astrocytomas, and between grade 2 + 3 and grade 4 IDH-mutant astrocytomas (adjusted *P* = 0.002, < 0.001, respectively).

**Table 1:** Summary of ADC_min_, ITSS and rCBV values among three grades of IDH-mutant astrocytomas

	Grade 2	Grade 3	Grade 4	Grade 2 vs 3	Grade 3 vs 4	Grade 2 vs 4
ADC_min_	1.17 ± 0.20	1.06 ± 0.21	0.74 ± 0.13	0.055	< 0.001	< 0.001
ITSS	0.43 ± 0.81	1.34 ± 1.40	2.55 ± 0.80	0.002	< 0.001	< 0.001
rCBV_max_	1.67 ± 0.85	2.11 ± 1.14	5.92 ± 2.26	0.506	< 0.001	< 0.001

*ADC*_*min*_ Minimum apparent diffusion coefficient, *ITSS* Intratumoral susceptibility signal, *rCBV*_*max*_ Relative maximum cerebral blood volume, *AUC* Area under the curve, *CI *Confidence interval

**Table 2:** Summary of ITSS levels among three grades of IDH-mutant astrocytoma

ITSS score	Grade 2	Grade 3	Grade 4	Total
0	40	13	0	53
1	11	4	4	19
2	2	1	2	5
3	3	11	16	30
Total	56	29	22	107

*ITSS *Intratumoral susceptibility signal

## Spearman’s analysis

Spearman’s test demonstrated significant relationships between the ITSS scores and the grades of tumor (*r* = 0.615, *P* < 0.001). Both ADC_min_ and rCBV_max_ have significant correlations with grades of IDH-mutant astrocytomas (rCBV_max_, *r* = 0.662 and *P* < 0.001; ADC_min_, *r* = 0.638 and *P* < 0.001).

## ROC curve analysis

**Fig. 5:**
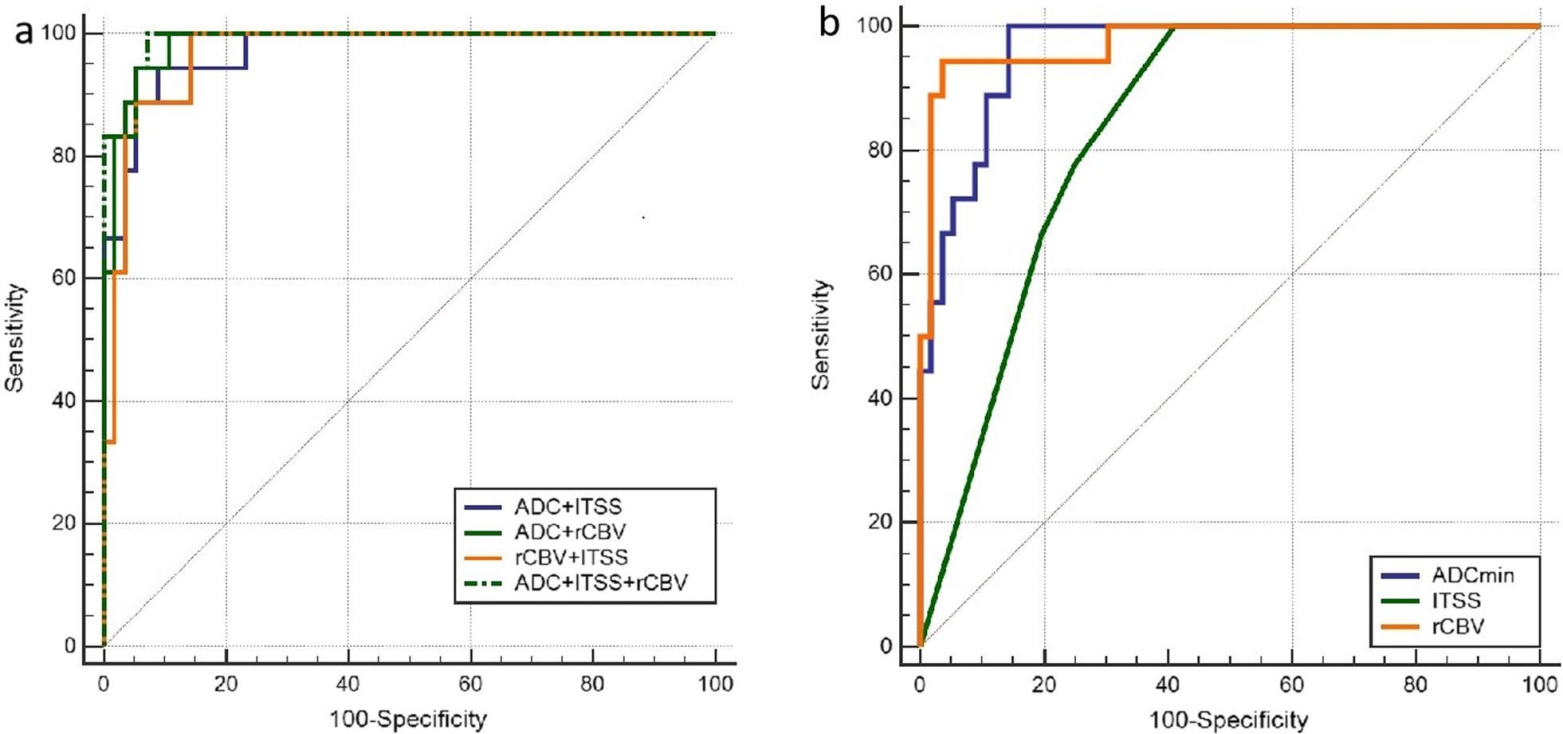
Comparison of receiver operating characteristic curves of ADC, ITSS and rCBV **a** and ADC + ITSS, ADC + rCBV, rCBV + ITSS and ADC + ITSS + rCBV **b** in differentiating grade 2 + 3 from grade 4 IDH-mutant astrocytomas

The results of ROC curve analysis are shown in Table 3–4 and Fig. 5. The AUCs of rCBVmax values in differentiating different grades of IDH mutant astrocytomas from grade 2 to grade 3, grade 3 to grade 4, grade 2 to grade 4,and grade 2 + 3 to grade 4 were 0.610, 0.955, 0.959 and 0.957, respectively. The AUCs of ADC_min_ in differentiating different grades of IDH mutant astrocytomas from grade 2 to grade 3, grade 3 to grade 4, grade 2 to grade 4,and grade 2 + 3 to grade 4 were 0.705, 0.907, 0.989 and 0.958, respectively. The AUCs of ITSS in differentiating different grades of IDH mutant astrocytomas from grade 2 to grade 3, grade 3 to grade 4, grade 2 to grade 4,and grade 2 + 3 to grade 4 were 0.683, 0.827, 0.946 and 0.903, respectively. ITSS > 1 was used to differentiate IDH-mutant astrocytomas grade 3 from grade 2 with sensitivity and specificity of 41% and 91%, respectively. The sensitivity and specificity of ITSS > 1 in differentiating grade 2 and grade 4 IDH-mutant astrocytomas were 81.8% and 58.6%, respectively. Compared grade 2 + 3 with grade 4 IDH-mutant astrocytomas, a threshod value of > 1 for ITSS resulted in a sensitivity of 81.8% and specificity of 80.0%. A rCBV cutoff value of 3.84 could differentiate grade 2 + 3 from grade 4 IDH-mutant astrocytomas with a sensitivity of 94.4% and specificity of 94.2%. Combination of rCBVmax, ADCmin and ITSS did not improve classification of grade 2 from grade 3 IDH-mutant astrocytomas. A significant difference was found on the AUC between ITSS and ADC_min_ + ITSS + rCBV_max_ (*Z* = 2.468, *P* = 0 0.014).

**Table 3:** Summary of ROC curve results to distinguish various grades of IDH-mutant astrocytoma

	Grade2vs3 (AUC,95%CI)	Grade 3 vs 4 (AUC, 95%CI)	Grade 2 vs 4 (AUC, 95%CI)	Grade 2 + 3 vs 4 (AUC, 95%CI)
ADC_min_	0.701(0.535–0.859)	0.898(0.804–0.991)	0.985(0.962–1.000)	0.956(0.906–0.993)
ITSS	0.678(0.537–0.816)	0.820(0.689–0.954)	0.943(0.885–1.000)	0.900(0.821–0.975)
rCBV_max_	0.602(0.456–0.758)	0.950(0.881–0.995)	0.953(0.898–1.000)	0.954(0.904–0.995)
ADC_min_ + ITSS	0.737(0.603–0.854)	0.921(0.788–0.983)	0.992(0.918–1.000)	0.967(0.935–1.000)
ADC_min_ + rCBV_max_	0.695(0.558–0.811)	0.985(0.962–1.000)	0.991(0.933–1.000)	0.973(0.964–1.000)
ITSS + rCBV_max_	0.704(0.545–0.841)	0.958(0.840–0.996)	0.960(0.946–1.000)	0.958(0.932–1.000)
ADC_min_ + ITSS + rCBV_max_	0.746(0.611–0.853)	0.987(0.886–1.000)	0.998(0.983–1.000)	0.994(0.983–1.000)

*AUC* Area under the curve, *CI* Confidence interval

**Table 4:** Threshold values, sensitivity, specificity, PPV and NPV for distinguishing grade 2 + 3 IDH-mutant astrocytoma from grade 4 IDH-mutant astrocytoma

	Cut-off	Sensitivity(%)	Specificity(%)	PPV(%)	NPV(%)
ADCmin	0.94	100.00	85.01	66.70	96.00
ITSS	1	81.80	80.00	51.40	94.40
rCBV_max_	3.84	94.44	94.64	85.00	98.10
ADC_min_ + ITSS		94.44	91.07	77.30	98.10
ADC_min_ + rCBV_max_		100.00	89.29	75.00	100.00
ITSS + rCBV_max_		100.00	85.71	69.20	100.00
ADC_min_ + ITSS + rCBV_max_		100.00	92.86	81.80	100.00

*PPV* Positive predictive value, *NPV* Negative predictive value

## Discussion

In the current study, we demonstrated the usefulness of ADC_min_, ITSS and rCBV_max_ vlaues for grading IDH-mutant astrocytomas. The combination of ADC_min_, ITSS and rCBV_max_ vlaues showed a better trend to distinguish the grade 2 and/or grade 3 IDH-mutant astrocytomas from grade 4 IDH-mutant astrocytomas.

The diffusion of water molecules could be limited by tumor cells and subcellular structures. DWI could noninvasively reflect the restricted diffusion of water molecules and evaluate tumor cellularity by ADC values [5]. Previous studies revealed that the ADC values decreased significantly in high-grade astrocytic tumors [14]. Our findings showed a significant difference of ADC values between grade 2 + 3 and grade 4, which were consistent with previous studies. In addition, our study demonstrated that the ADC_min_ value of grade 4 IDH-mutant astrocytomas was comparable with study by Tan et al. [15]. Prior studies reported the differences of ADC values among grade 2, 3 and 4 astrocytoma without considering IDH mutational status [16]. However, the current study assessed the values of ADC_min_ in different grades of IDH-mutant astrocytomas. Moreover, we also evaluated the difference of grade 2 + 3 and grade 4 IDH-mutant astrocytomas since some researchers have reported similar prognosis betweem grade 2 and grade 3 IDH-mutant astrocytomas [17–19]. Consequently, we found significant difference of ADC_min_ values between grade2 + 3 and grade 4 tumors, but no significant difference of ADCmin was abserved between grade 2 and grade 3 tumors. It has been reported that higg ADC value was correlated with favorable prognosis [20], our results will be conducive to the prognosis evaluation of patients with IDH-mutant astrocytomas based on ADC measurement.

DSC-PWI could provide the vascularity and angiogenesis information of astrocytoma by rCBV values [21]. Higher grade gliomas tend to have higher rCBV values [6, 22, 23]. Several studies have reported that rCBV was useful in grading astrocytomas [24–26]. Saini et al. [10] found that CBV could be used to differentiate the grade II gliomas and grade III gliomas with high sensitivity and specificity. In contrast, our study found no significant difference of rCBV_max_ between grade 2 and grade 3. This discrepancy may be due to the enrolled patients were different and previous investigations did not consider the IDH mutational status of gliomas. It has been shown that IDH mutation leads to 2-HG accumulation, which may decreased hypoxia-inducible-factor-1 activation and inhibited angiogenesis-related signaling [27, 28]. Some previous studies reported that rCBV values were significantly correlated with time to progression in patients with gliomas [20, 29, 30]. This may also be used to explain why grade 2 and grade 3 IDH-mutant astrocytomas have few difference in survival. We found that the rCBV_max_ values of grade 2 and grade 3 were lower than those of grade 4, which may be ascribed to elevate vascular proliferation in high-grade tumors.

Previous studies have demonstrated that SWI was useful for grading astrocytomas [9, 10]. In our study, most of patients with grade 4 IDH-mutant astrocytomas demonstrated an ITSS score of 3 and majority of patients with grade 2 showed no ITSS. Astrocytomas contained high levels of deoxyhemoglobin, due to angiogenesis and the increase of blood supply [24]. Additionally, deoxyhemoglobin generates susceptibility effect, which is related to the signal loss on SWI. The present study showed that ITSS was not detected in 13 of 29 patients with grade 3 IDH-mutant astrocytomas. However, Saini et al. [10] reported that all of the anaplastic astrocytomas could be seen with ITSS. A possible reason was the relatively small sample of anaplastic astrocytoma in their study. Our findings revealed that ITSS scores were significant difference between grade 2 + 3 and grade 4 IDH-mutant astrocytomas, suggesting that ITSS may serve as a potential biomarker for grading IDH-mutant astrocytic gliomas. In the current study, ITSS socres were significant different between grade 2 and grade 3 IDH-mutant astrocytomas while rCBV_max_ were not. We speculated that ITSS reflected not only blood vessels but also micro-bleeding and necrosis of tumor, which may lead to the above results.

We found a positive correlation between rCBVmax and tumor grade, which is consistent with previous study [9]. Our study found that ITSS was correlated with tumor grade; this finding was in accordance with the results from previous studies [9, 31]. Our results showed that there was a negative correlation between ADC_min_ and tumor grade,suggesting that tumor grade is closely related to the proliferation of tumor cells [32].

We found that the area under ROC for combined ADC_min_, rCBV_max_ and ITSS was significantly larger than ITSS used alone for grade 3 and grade 4 IDH-mutant astrocytomas. For grade 2 + 3 and grade 4 IDH-mutant astrocytomas, AUC for combined DWI, SWI and DSC-PWI was larger than DWI, SWIand DSC-PWIalone. Our results suggested that the combination of those advanced MRI techniques could improve the diagnostic efficacy in grading IDH-mutant astrocytic gliomas.

This study has some limitations. The retrospective design and single-center sampling may lead to selection bias. Wetzel et al. [33]assessed inter- and intraobserver reproducibility for different techniques of measuring rCBV in patients with intracranial mass lesions, and they found the method that we used to obtain rCBV provides a high interobserver and intraobserver reproducibility. For this retrospective analysis,the DSC-PWI acquisition in our study is suboptimal and was not obtained according to the national consensus recommendation [34]. A multi-centered prospective investigation with standard DSC-PWI acquisition is warranted to verify these results and ensure the reproducibility. Additionally, other genetic alterations such as CDKN2A/B and CDK4 were not assessed because of relevant molecular informations were not avaliable in our study. Advanced MRI imaging techniques combined with larger datasets on other genetic alterations should be performed in future studies.

## Conclusions

DWI, SWI and DSC-PWI are useful for assessing the tumor grade in this cohort of patients with IDH-mutant astrocytomas. A combination of ADC_min_, ITSS and rCBV_max_ may improve the diagnostic accuracy of IDH-mutant astrocytomas grading.

## Data Availability

Due to statutory provisions regarding data and privacy protection, the dataset supporting the conclusions of this article is available upon individual request directed. to the corresponding author.

## Abbreviations

MRI: Magnetic resonance imaging
CBV: Cerebral blood volume
rCBV_max_: Relative maximum cerebral blood volume
ADC_min_: Minimum apparent diffusion coefficients
DWI: Diffusion weighted imaging
SWI: Susceptibility-weighted imaging
ITSS: Intratumoral susceptibility signal intensity
ADC: Apparent diffusion coefficients
DSC-PWI: Dynamic susceptibility contrast perfusion-weighted imaging
FOV: Field of view
ROI: Region of interest
